# Disposable Injection Molded Conductive Electrodes Modified with Antimony Film for the Electrochemical Determination of Trace Pb(II) and Cd(II)

**DOI:** 10.3390/s19214809

**Published:** 2019-11-05

**Authors:** Savvina Christidi, Alexia Chrysostomou, Anastasios Economou, Christos Kokkinos, Peter R. Fielden, Sara J. Baldock, Nicholas J. Goddard

**Affiliations:** 1 Department of Chemistry, National and Kapodistrian University of Athens, 157 71 Athens, Greece; 2 Department of Chemistry, Lancaster University, Lancaster LA1 4YB, UK; 3 Process Instruments (UK) Ltd., March Street, Burnley BB12 0BT, UK

**Keywords:** injection molding, antimony film, electrode, stripping voltammetry, Cd(II), Pb(II)

## Abstract

This work describes a novel electrochemical sensor fabricated by an injection molding process. This device features a conductive polymer electrode encased in a plastic holder and electroplated in situ with a thin antimony film. The antimony film sensor was applied to the determination of Pb(II) and Cd(II) by anodic stripping voltammetry (ASV). The deposition of Sb on the sensor was studied by cyclic voltammetry (CV) and microscopy. The experimental variables (concentration of the antimony plating solution, deposition potential and time, stripping waveform) were investigated, and the potential interferences were studied and addressed. The limits of detection were 0.95 μg L^−1^ for Pb(II) and 1.3 for Cd(II) (at 240 s of preconcentration) and the within-sensor percentage relative standard deviations were 4.2% and 4.9%, respectively, at the 25 μg L^−1^ level (*n* = 8). Finally, the sensor was applied to the determination of Pb(II) and Cd(II) in a phosphorite sample and a lake water sample.

## 1. Introduction

Heavy metals, such as Cd and Pb, are highly toxic species that can find their way in organisms via the consumption of food and water, breathing, and absorption through the skin [1]. Different variants of spectroscopic techniques are mainly used for the determination of heavy metals in various matrices [1,2,3]. Stripping analysis has been established as an important alternative technique for trace metal determination due to its high sensitivity, simple, low-cost, and portable instrumentation, and scope for on-site analysis [4,5,6]. Mercury electrodes (mainly the hanging mercury drop electrode (HMDE) and the mercury film electrode (MFE)) exhibit unique characteristics that make them excellent candidates for trace metal assays by stripping analysis. Yet, mercury and it vapors are toxic, and the relevant legislation has imposed severe restrictions on the use, transportation, and handling of mercury. Therefore, many efforts have been made to develop more environment-friendly and “green” electrode materials as replacements for mercury electrodes [7,8].

Since the landmark introduction of the bismuth film electrode (BiFE) in 2000 [9], Bi electrodes have been widely and successfully used in a host of electroanalytical applications [10,11]. The antimony film electrode (SbFE), consisting of a thin film of antimony on a glassy carbon support, was reported in 2007 as another useful and environment-friendly electrode material [12]. The main advantages of Sb electrodes are their low overpotential for hydrogen evolution even in acidic media, and the well-suppressed Sb stripping current, while their overall performance in ASV approaches that of bismuth electrodes, as illustrated in a recent comprehensive review of this subject [13].

Injection molding is a universal procedure for the fast and low-cost fabrication of plastic objects in different configurations. Electrodes made of conductive thermoplastic materials have been used in the past for the ASV determination of Cu(II) [14] and Pb(II) [15], but these electrodes were used directly as prepared (i.e., without any modification step prior to analysis). However, such unmodified electrodes have limitations in terms of sensitivity as well as in terms of the range and number of co-detectable target metals, so that the aforementioned applications have been limited to the detection of single metal cations [14,15].

In the present work, we describe the fabrication of a new disposable electrochemical sensor fabricated by an injection molding process. The proposed configuration (a conductive polymer electrode encased in a plastic holder) greatly facilitates the mass fabrication of disposable and reproducible sensors in a rapid manner and at low cost. During the analysis, the electrode is electroplated in situ with a thin film of antimony, thereby enabling the simultaneous determination of Pb(II) and Cd(II) by anodic stripping voltammetry (ASV) with markedly higher sensitivity than on bare electrodes.

## 2. Materials and Methods

### 2.1. Chemical and Reagents

All the chemicals were of analytical grade and purchased from Merck (Darmstadt, Germany) or Sigma-Aldrich (St. Louis, MO, USA). Doubly distilled water was used throughout. Stock solutions containing 10 and 100 mg L^−1^ of different metals (Cd(II), Pb(II), Sn(II), Zn(II), In(III), Cu(II), As(III) and Tl(I)) were prepared from 1000 mg L^−1^ standard solutions after appropriate dilution with water. Direct additions of a 1000 mg L^−1^ standard solution of Sb(III) in the sample were made for the in situ formation of the antimony film. The working supporting electrolyte was 0.01 mol L^−1^ HCl.

The phosphorite sample was provided by a local company producing fertilizers, and is being used as a raw material for the production of phosphate fertilizers. The lake water sample was provided by the Laboratory of Environmental Chemistry of our Department.

### 2.2. Instrumentation

Electrochemical experiments were performed with a Palmsens potentiostat controlled by the PSTrace 4.1 software (Palm Sens BV, Houten, The Netherlands). The potentiostat was connected to the injection molded sensor (working electrode), an Ag|AgCl|3M KCl reference electrode, and a Pt-wire counter electrode with crocodile clips. The sample was placed in a glass cell equipped with a stirring bar rotating at approximately 300 rpm by means of a magnetic stirrer.

The surface of the microdisc electrode was inspected by means of an optical microscope (Olympus MX51-F, Olympus Corporation, Tokyo, Japan).

A Perkin Elmer SIMAA 6000 multi-elemental atomic absorption spectrometer with a THGA graphite furnace, and Zeeman background correction (The Perkin Elmer Corporation, Norwalk, CT, USA) was used for comparison purposes.

### 2.3. Fabrication of the Sensor

**Figure 1:**
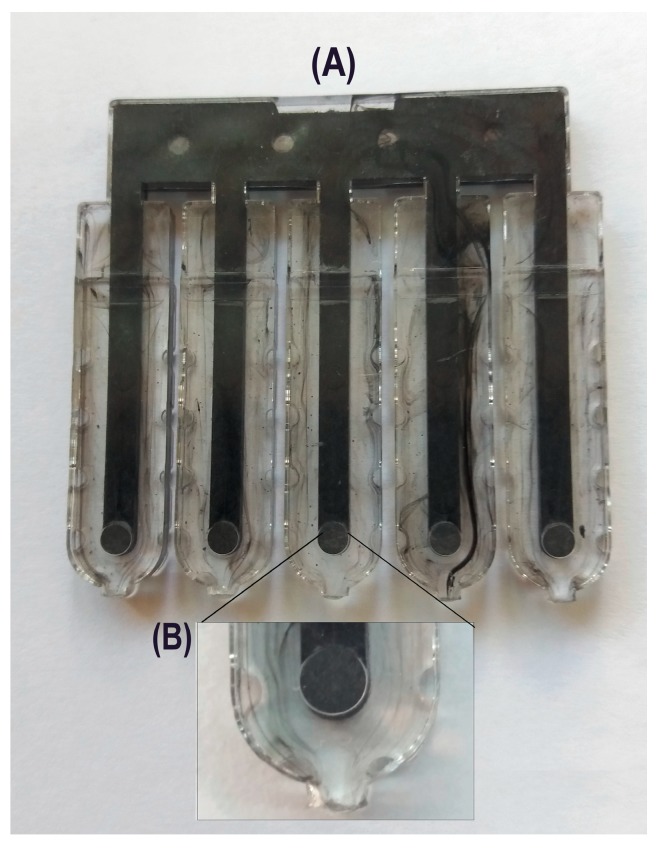
(**A**) Photograph of a batch of five injection molded sensors; (**B**) Magnification of the active sensing area of a single sensor.

The electrodes were injection molded from a conducting polymer material (40% carbon fiber-filled high-impact polystyrene (HIPS), RTP 487, RTP Company (UK) Plastics Ltd., Bury, UK), using an injection molder (Babyplast 6/6, Cronoplast SA, Barcelona, Spain) in sets of 5 (Figure 1A). Mold tools were fabricated from brass bar stock using a milling machine (CAT3DM6 CNC, Datron Technology Ltd., Milton Keynes, UK). The molding conditions were as follows: plasticizing temperature 230 °C; injection chamber temperature 220 °C; injection nozzle temperature 200 °C; injection pressure 50 bar; injection time 6 s; and cooling time 12 s. The conducting electrodes were first fabricated and placed into a holder made of clear non-conductive polystyrene (Northern Industrial Plastics Ltd., Chadderton, UK) using an overmolding process. The sensors measured 1 cm × 1 cm × 0.4 cm. The whole area of the conductive electrodes was covered by the holder except for a disk-shaped area with a diameter of 2.5 mm, which was the active surface of the sensor (Figure 1B).

### 2.4. Experimental Procedure

The potential window of the sensor was studied in 0.01 mol L^−1^ HCl solution by cyclic volatmmetry (CV) by scanning the potential from −1.2 V to +0.5 V (versus Ag/AgCl) and reversely with a scan rate of 50 mV s^−1^.

For ASV measurements, the solution was spiked with 2 mg L^−1^ Sb(III) (and with the appropriate concentration of the target metals as required), and co-deposition of the antinomy film and the analytes was carried out at −1.2 V for 240 s under stirring. Then, the solution was allowed to equilibrate in static solution for 10 s and the stripping step was performed by the scanning the potential in the range from −1.0 V to +0.2 V in the square wave (SW) mode (frequency, 25 Hz; pulse height, 25 mV; step, 4 mV). Finally, a cleaning step at +0.2 V for 30 s under stirring was applied to oxidize the antimony film and any remaining target metals on the electrode.

## 3. Results and Discussion

### 3.1. Deposition of Sb on the Electrode

**Figure 2:**
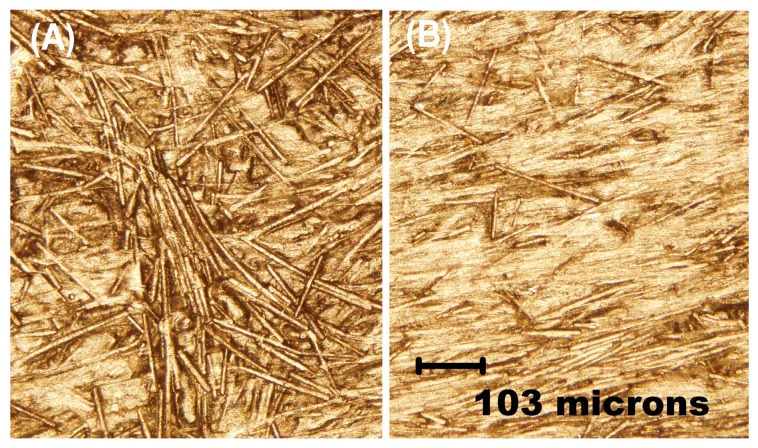
Photographs of the surface morphology of: (**A**) a bare conductive electrode; (**B**) an Sb-coated conductive electrode.

Optical microscopy of the bare electrode’s surface reveals the individual carbon fibers embedded in the polystyrene matrix (Figure 2A). After coating the surface with an antimony film, the carbon fibers are almost completely covered by the metal coating (Figure 2B). This study indicates that an Sb film was successfully deposited on the surface of the conductive electrode.

**Figure 3:**
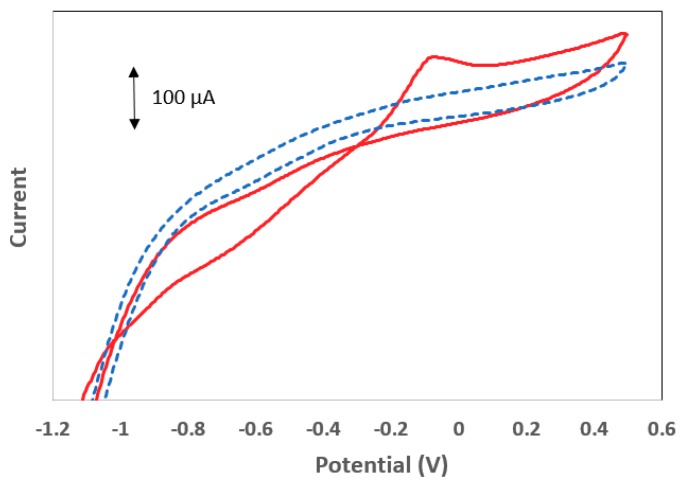
Cyclic voltammetry (CV) in the range from −1.1 V to +0.5 V preceded by an accumulation step at –1.2 V for 300 s in 0.01 mol L^−1^ HCl (dotted blue line) and after the addition of 100 mg L^−1^ of Sb(III) (red solid line).

The deposition of Sb on the electrode surface was further studied by CV in the range from −1.1 V to +0.5 V preceded by an accumulation step at –1.2 V for 300 s (Figure 3). In a solution containing only the supporting electrolyte (Figure 3, dotted line), no oxidation or reduction peaks were observed. After the addition of 100 mg L^−1^ of Sb(III) (Figure 3, solid line), a peak at −0.06 V appeared in the forward anodic scan, which was due to the oxidation of Sb deposited during the accumulation step. The reverse cathodic scan did not exhibit an Sb reduction wave, which was attributed to the poor electrochemical reversibility of Sb. Moreover, a crossover of the forward and reverse scans was observed at –0.29 V; this effect is characteristic of a metal deposition mechanism described by nucleation and growth, in which the initial formation of Sb nuclei on the electrode surface increases the rate of further deposition [16].

It must be noted than no Sb oxidation signal was observed in the voltammogram when concentrations of Sb(III) lower than 40 mg L^−1^ were used. This effect was also evident in the stripping voltammetry experiments in the following sections (which involved Sb(IIII) concentrations ≤4 mg L^−1^) in which no Sb stripping peak was observed. The presence of very weak Sb stripping peaks, or the compete absence of an Sb stripping signal, have been well documented and studied in the literature, but this phenomenon has not been conclusively accounted for so far [12,17,18,19].

### 3.2. Selection of the Chemical and Instrumental Conditions

In the following experiments, a 0.01 mol L^−1^ HCl solution was used as the supporting electrolyte as proposed earlier [12,13]. The study of the working conditions involved: the concentration of the Sb(III) solution; the deposition potential; the deposition time; and the voltammetric stripping mode.

**Figure 4:**
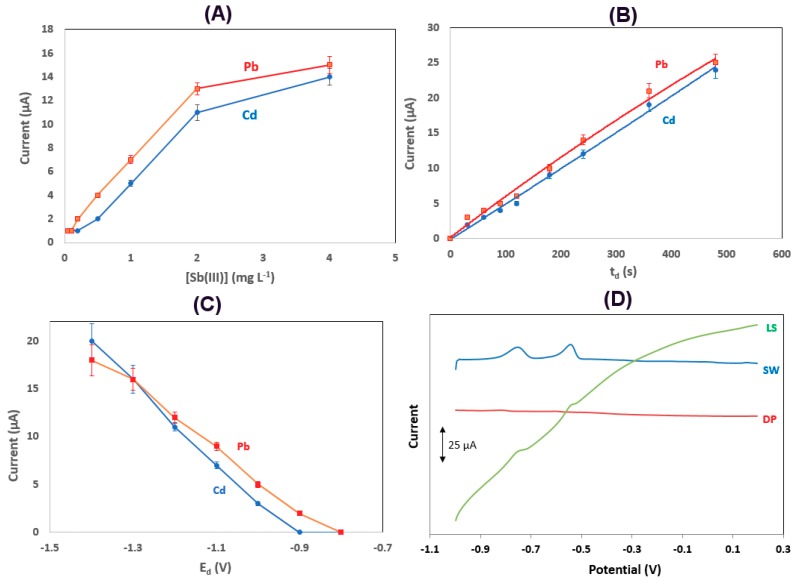
Study of (**A**) the Sb(III) concentration; (**B**) the deposition time; (**C**) the deposition potential; (**D**) the voltammetric scan waveform (voltammograms are offset for clarity). Conditions: 25 μg L^−1^ of Cd(II) και Pb(II); Sb(III) concentration, 20 mg L^−1^; supporting electrolyte, 0.01 mol L^−1^ HCl; deposition potential, −1.2 V; deposition time, 240 s.

The effect of the Sb(III) concentration used for the in situ formation of the Sb film on the stripping peak currents of Pb and Cd is illustrated in Figure 4A: both signals increased rapidly with increasing Sb(III) concentration up to 2 mg L^−1^ and started to level off at higher Sb(III) concentrations. An Sb(III) concentration of 2 mg L^−1^ was selected as a compromise between sufficient sensitivity, low reagent consumption, and waste generation. This study shows that the Sb-modified conductive electrode exhibits markedly higher sensitivity than the bare unmodified electrode.

The effect of the deposition time on the stripping peak currents of Pb and Cd is illustrated in Figure 4B, indicating an almost linear increase in both signals with the deposition time in the range examined (0–480 s). A deposition time of 240 s was normally used as a trade-off between short analysis time and sufficient sensitivity (unless exceptional sensitivity was required).

The effect of the deposition potential on the stripping peak currents of Pb and Cd is illustrated in Figure 4C. In this case, the Pb and Cd signals increased as the deposition potential became more negative, suggesting higher deposition efficiency at more cathodic deposition potentials. However, at potentials more negative than −1.2 V, the repeatability deteriorated, which was probably due to the generation of H_2_ bubbles on the surface of the electrode that interfered with the deposition process; therefore, a deposition potential of −1.2 V was selected.

Finally, a comparison was made between the linear sweep (LS), differential pulse (DP), and SW stripping modes (Figure 4D). The LS mode produced high background current, while the sensitivity of the DP mode was very low. The SW modulation was the most satisfactory, combining adequate sensitivity and a flat baseline.

### 3.3. Metrological Features

**Figure 5:**
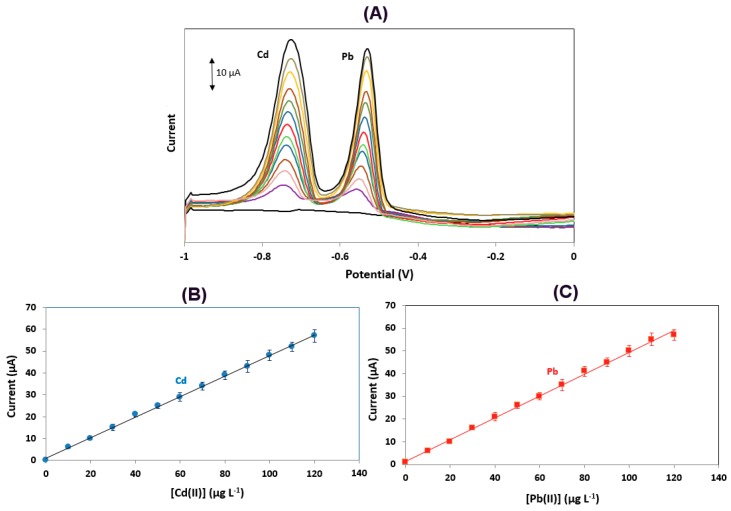
(**A**) Voltammograms for the determination of Pb(II) and Cd(II) in the range of 0–120 μg L^−1^; (**B**) calibration plot for Cd(II); (**C**) calibration plot for Pb(II). Conditions as in Figure 4.

Calibration for Cd(II) and Pb(II) was performed in the concentration range 0–120 μg L^−1^. The calibration features and metrological attributes (calibration equation, coefficient of determination, and limits of detection) for Pb(II) and Cd(II) are summarized in Table 1, while representative voltammograms and calibration plots are illustrated in Figure 5. The limits of detection obtained with the proposed sensor were competitive with existing SbFEs using various other support materials [13].

The within-sensor repeatability was calculated by performing eight repetitive measurements of a solution containing 25 μg L^−1^ l of the target metals at the same sensor; the percentage relative standard deviations were 4.2% and 4.9% for Pb(II) and Cd(II), respectively. The between-sensor reproducibility was estimated by performing measurements of a solution containing 25 μg L^−1^ l of the target metals at five different sensors randomly selected from different batches; the % relative standard deviations were 9.5% and 11.1% for Pb(II) and Cd(II), respectively. In addition, each sensor could be used for several tens of measurements without a statistical loss of sensitivity.

### 3.4. Interferences

**Figure 6:**
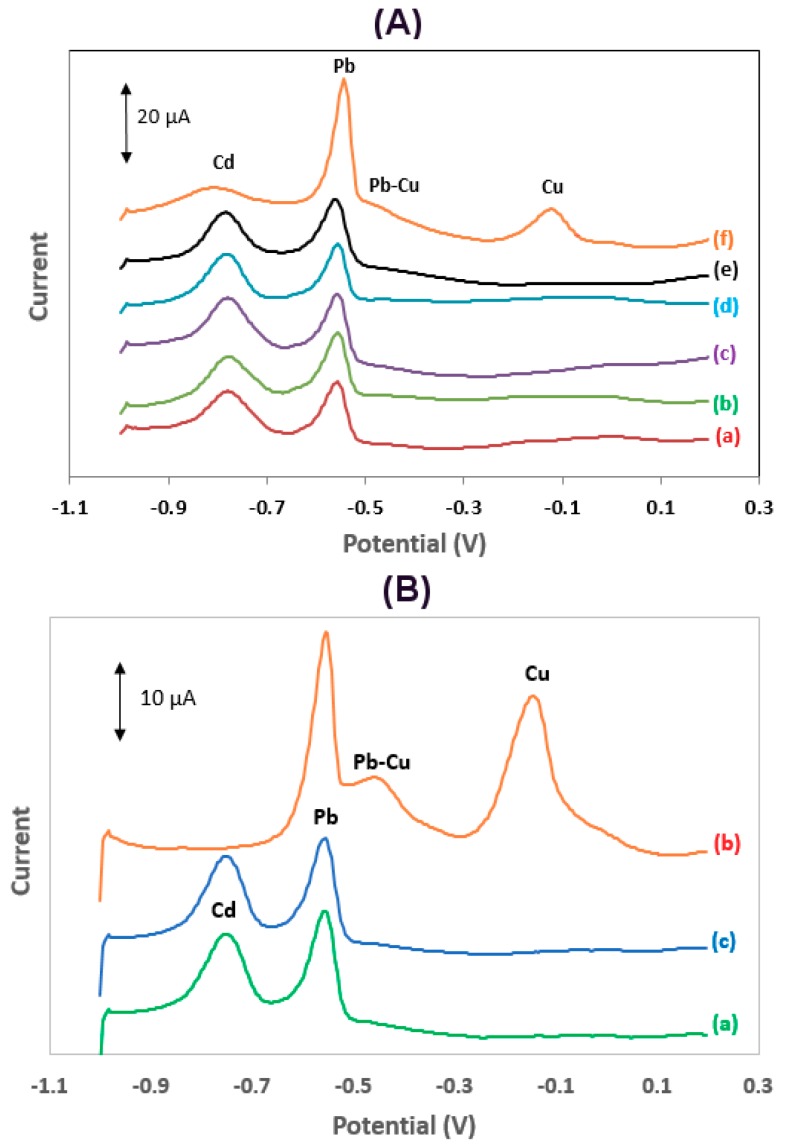
(**A**) Voltammograms for the determination of 25 μg L^−1^ of Pb(II) and Cd(II) (**a**) before, and after the successive addition of 50 μg L^−1^ of: (**b**) Sn(II), (**c**) Zn(II), (**d**) Tl(I) + In(III), (**e**) Hg(II) + As(III), and (**f**) Cu(II); (**B**) (**a**) voltammograms for the determination of 25 μg L^−1^ Pb(II) and Cd(II), (**b**) after the addition of 150 μg L^−1^ Cu(II), (**c**) as (b) after the addition of 1.0 × 10^−5^ mol L^−1^ Κ_4_[Fe(CN)_6_] (voltammograms are offset for clarity). Conditions as in Figure 4.

The effect of common interferents (Sn(II), Zn(II), TI(I), In(III), Cu(II), Hg(II) and As(III)) on the determination of Cd(II) and Pb(II) was studied. As illustrated in Figure 6Af, only Cu(II) interfered, causing a significant increase in the Pb stripping signal, a suppression of the Cd stripping signal, the appearance of a stripping Cu signal at −0.13 V, and the appearance of a new peak at −0.45 V (manifesting as a bump on the anodic side of the Pb peak and more clearly defined at higher Cu(II):Pb(II) concentration ratios (Figure 6Bb). This latter peak has been observed in various earlier previous studies at carbon [16,20,21] and antimony electrodes [22], and has been attributed to the oxidation of a Pb–Cu intermetallic compound. The Cu(II) interference was effectively eliminated using ferrocyanide anions to selectively mask Cu(II), as proposed earlier [23,24] (Figure 6B).

## 4. Application

**Figure 7:**
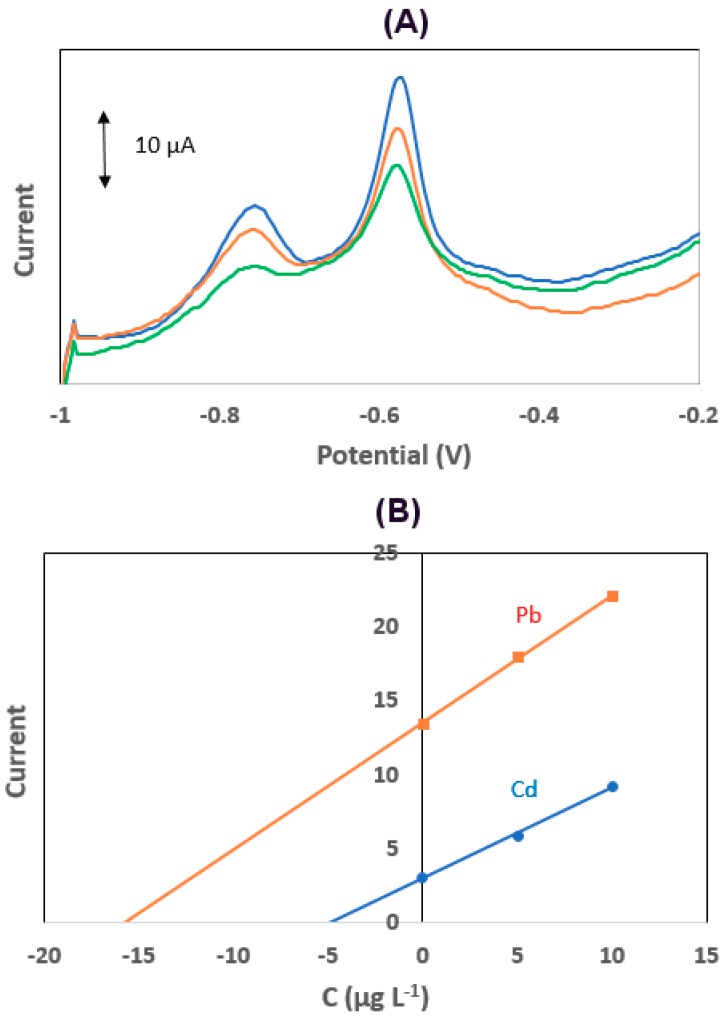
(**A**) Voltammograms for the determination of Pb(II) and Cd(II) in a phosphorite sample; (**B**) standard additions plots. Conditions as in Figure 4; the sample was spiked with 2.0 × 10^−5^ mol L^−1^ Κ_4_[Fe(CN)_6_] before the analysis.

Cd and Pb were determined in a phosphorite sample and a lake water sample using the detection protocol described in Section 2.4 spiking the samples with 2.0 × 10^−5^ mol L^−1^ Κ_4_[Fe(CN)_6_] before the analysis. Phosphorites are the raw materials used for the production of phosphate fertilizers, and their content in heavy metals should be closely monitored since this has an impact on potential agricultural contamination with heavy metals [25,26,27]. For the digestion of the phsophorite sample, 0.25 g of sample was treated with 10 mL of concentrated HNO_3_ in a microwave digester (CEM Mars 5, CEM Corporation, Matthews, NC, USA). The digested sample was boiled to dryness, after which the residue was dissolved in 5 mL of 0.1 mol L^−1^ HCl, transferred to a 50-mL volumetric flask, and brought to the mark with doubly distilled water. The quantification of Pb(II) and Cd(II) was performed by the method of multiple standard additions. Voltammograms of the digested sample and standard additions plots are illustrated in Figure 7A,B, respectively. The Cd and Pb content in the sample was 0.97 ± 0.08 and 3.2 ± 0.4 mg Kg^−1^ (*n* = 3), respectively, while the reference values obtained by atomic absorption spectrometry were 1.04 ± 0.09 and 3.0 ± 0.5 mg Kg^−1^. Therefore, no statistical difference (at the 2σ level) between the two methods existed. The lake water sample was filtered and acidified (by adding 1 mL of 0.1 mol L^−1^ HCl in 9 mL of the sample). Preliminary analysis indicated that the Pb(II) and Cd(II) concentrations were lower than the LOD of the method, and the accuracy was estimated by recoveries in the sample spiked with 10 μg L^−1^ of Pb(II) and Cd(II) and quantification by the method of multiple standard additions; the average recoveries were 98 ± 3% (*n* = 3) for Pb(II) and 97 ± 4% (*n* = 3) for Cd(II), which were deemed satisfactory for routine analysis.

## 5. Conclusions

In this work, a novel electrochemical sensor is described that features a conductive polymer electrode encased in a plastic holder. The sensor has been applied to the ASV determination of Pb(II) and Cd(II) after in situ modification with an Sb film. The formation of the Sb layer on the electrode surface was studied by CV and optical microscopy. The sensor enabled limits of detection for the target metals in the low μg L^−1^ level, providing satisfactory precision and scope for use in a semi-disposable mode. The only serious interference was Cu(II), which was alleviated by the addition of ferrocyanide in the sample. The favorable analytical performance, the extremely low cost, the easy surface modification with an Sb film, as well as the scope for rapid mass fabrication make these devices very promising for on-site monitoring purposes.

## Figures and Tables

**Table 1:** Calibration features for Pb(I) and Cd(II).

	Calibration Equation	R ^1^	LOD (μg L^−^^1^) ^2^
Cd(II)	I_p_ (μΑ) = (0.469 ± 0.004) C_Cd_ (μg L^−1^) + (0.347 ± 0.103) (μΑ)	0.998	1.3
Pb(II)	I_p_ (μΑ) = (0.482 ± 0.006) C_Pb_ (μg L^−1^) + (0.442 ± 0.152) (μΑ)	0.997	0.95

^1^ Coefficient of determination. ^2^ Limit of detection (LOD).

## References

[1] Bhattacharjee T., Goswami M. (2018). Heavy Metals (As, Cd & Pb) Toxicity & Detection of These Metals in Ground Water Sample: A Review on Different Techniques. Int. J. Eng. Sci. Inven..

[2] Bulska E., Ruszczyńska A. (2017). Analytical Techniques for Trace Element Determination. Phys. Sci. Rev..

[3] Helaluddin A.B.M., Saadi Khalid R., Alaama M., Atif Abbas S. (2016). Main Analytical Techniques Used for Elemental Analysis in Various Matrices. J. Pharm. Res..

[4] Economou A., Kokkinos C., Arrigan D.W.M. (2016). Advances in stripping analysis of metals. Electrochemical Strategies in Detection Science.

[5] Wang J. (1985). Stripping Analysis: Principles, Instrumentation and Applications.

[6] Wang J., Tian B., Wang J., Lu J., Olsen C., Yarnitzky C., Olsen K., Hammerstrom D., Bennett W. (1999). Stripping analysis into the 21st century: Faster, smaller, cheaper, simpler and better. Anal. Chim. Acta.

[7] Arino C., Serrano N., Díaz-Cruz J.M., Esteban M. (2017). Voltammetric determination of metal ions beyond mercury electrodes. A review. Anal. Chim. Acta.

[8] Alves G.M.S., Rocha L.S., Soares H.M.V.M. (2017). Multi-element determination of metals and metalloids in waters and wastewaters, at trace concentration level, using electroanalytical stripping methods with environmentally friendly mercury free-electrodes: A review. Talanta.

[9] Wang J., Lu J., Hocevar S.B., Farias P.M.A., Ogorevc B. (2000). Bismuth-coated carbon electrodes for anodic stripping voltammetry. Anal. Chem..

[10] Švancara I., Prior C., Hočevar S.B., Wang J. (2010). A decade with bismuth-based electrodes in electroanalysis. Electroanal..

[11] Kokkinos C., Economou A. (2008). Stripping analysis at bismuth-based electrodes. Curr. Anal. Chem..

[12] Hocevar S.B., Švancara I., Ogorevc B., Vytřas K. (2007). Antimony film electrode for electrochemical stripping analysis. Anal. Chem..

[13] Serrano N., Díaz-Cruz J.M., Arino C., Esteban M. (2016). Antimony-based electrodes for analytical determinations. TrAC.

[14] Gharib Naseri N., Baldock S.J., Economou A., Goddard N.J., Fielden P.R. (2008). Disposable Injection-Moulded Cell-on-a-Chip Microfluidic Devices with Integrated Conducting Polymer Electrodes for On-LineVoltammetric and Electrochemiluminescence Detection. Electroanalysis.

[15] Kokkinos C., Economou A., Goddard N.J., Fielden P.R., Baldock S.J. (2016). Determination of Pb(II) by sequential injection/stripping analysis at all-plastic electrochemical fluidic cells with integrated composite electrodes. Talanta.

[16] Prado C., Wilkins S.J., Marken F., Compton R.G. (2002). Simultaneous Electrochemical Detection and Determination of Lead and Copper at Boron-Doped Diamond Film Electrodes. Electroanalysis.

[17] Sebez B., Ogorevc B., Hocevar S.B., Veber M. (2013). Functioning of antimony film electrode in acid media under cyclic and anodic stripping voltammetry conditions. Anal. Chim. Acta.

[18] Guzsvany V., Nakajima H., Soh N., Nakano K., Imato T. (2010). Antimony-film electrode for the determination of trace metals by sequential-injection analysis/anodic stripping voltammetry. Anal. Chim. Acta.

[19] Guzsvany V., Nakajima H., Soh N., Nakano K., Svancara I., Vytras K., Bjelica L., Imato T. (2011). Anodic stripping voltammetry combined with sequential injection analysis for measurements of trace metal ions with bismuth- and antimony film electrodes under comparable conditions. Electroanalysis.

[20] Agra-Gutiérrez C., Hardcastle J.L., Ball J.C., Compton R.G. (1999). Anodic stripping voltammetry of copper at insonated glassy carbon-based electrodes: Application to the determination of copper in beer. Analyst.

[21] Dragoe D., Spătaru N., Kawasaki R., Manivannan A., Spătaru T., Tryk D.A., Fujishima A. (2006). Detection of trace levels of Pb^2+^ in tap water at boron-doped diamond electrodes with anodic stripping voltammetry. Electrochim. Acta.

[22] Slavec M., Hocevar S.B., Baldrianova L., Tesarova E., Svancara I., Ogorevc B., Vytras K. (2010). Antimony Film Microelectrode for Anodic Stripping Measurementof Cadmium(II), Lead(II) and Copper(II). Electroanalysis.

[23] Yang D., Wang L., Chen Z., Megharaj M., Naidu R. (2013). Investigation of Copper(II) Interference on the Anodic StrippingVoltammetry of Lead(II) and Cadmium(II) at Bismuth Film Electrode. Electroanalysis.

[24] Xiao L., Zhou S., Hu G., Xu H., Wang Y., Yuan Q. (2015). One-step synthesis of isoreticular metal–organic framework-8 derived hierarchical porous carbon and its application in differential pulse anodic stripping voltammetric determination of Pb(II). RSC Adv..

[25] Serrini G., Muntau H., Colinet E., Griepink B. (1983). Certification of the Contents of As, B, Cd, Cr, Co, Cu, Mn, Hg, Ni, Ti, V and Zn in a Natural Moroccan Phosphate Rock. Fresenius Z. Anal. Chem..

[26] Maroulis M., Economou A., Voulgaropoulos A. (2007). Determination of Cd and Pb in Phosphorites and PhosphateFertilizers by Means of a Portable Voltammetric Analyzer Based on“Virtual Instrumentation”. Electroanalysis.

[27] Voulgaropoulos A., Paneli M., Papaefstathiou E., Stavroulias S. (1991). Comparative determinations of cadmium and lead in phosphorites dissolved in nitric acid and aqua regia using differential pulse anodic stripping voltammetry and atomic absorption spectrophotometry. Fres. J. Anal. Chem..

